# Does medial support decrease major complications of unstable proximal humerus fractures treated with locking plate?

**DOI:** 10.1186/1471-2474-14-102

**Published:** 2013-03-22

**Authors:** Woo-Bin Jung, Eun-Sun Moon, Sung-Kyu Kim, David Kovacevic, Myung-Sun Kim

**Affiliations:** 1 Department of Orthopaedic Surgery, Chonnam National University College of Medicine, 671, Jebong-Ro, Dong-Gu, Gwangju, 501-757, South Korea; 2 Department of Orthopaedic Surgery, Cleveland Clinic Foundation, Cleveland, OH, USA

**Keywords:** Proximal humerus fracture, Locking plate, Complication, Medial support, Osteoporosis

## Abstract

**Background:**

The purpose of this study was to evaluate the role of medial support and clinical factors responsible on outcomes and major complications associated with treatment of unstable proximal humerus fractures using a locking plate and suture augmentation.

**Methods:**

Sixty-three cases in 62 patients (42 female, 20 male) were evaluated between September 2004 and October 2008. Cases were divided into either a medial support group (36 cases) or non-medial support group (27 cases). Clinical and radiographic evaluations included Neer’s evaluation criteria, the neck-shaft angle using the Paavolainen method, and complications. We analyzed the correlation between bone- and fracture- related complications and three independent clinical variables, such as the presence of medial support, fracture type, and osteoporosis by way of multivariate logistic regression.

**Results:**

There were statistically significant differences in the overall incidence of complications based on the presence of medial support (p = 0.014) and preoperative fracture type (p = 0.018), but no differences based on the presence of osteoporosis (p = 0.157). According to multivariate logistic regression analysis, the restoration of medial support was the most reliable factor to prevent bone- and fracture- related complications. In addition, when we compared the incidence of bone- and fracture-related complications in the presence or absence of medial support among 30 patients with osteoporosis, the group with restoration of medial support had only one complication of humeral head osteonecrosis despite the presence of osteoporosis (5.9% vs. 46.2%, p = 0.025). According to Neer’s criteria, excellent or satisfactory clinical results accounted for seventy-three percent of the total cases (46 of 63 cases). Seventy-eight percent (49 of 55 cases) showed good radiographic results by the Paavolainen method. There were 14 complications in 13 of 63 cases (20.6%).

**Conclusions:**

In the treatment of unstable proximal humerus fractures with locking plate technology and suture augmentation, we suggest that obtaining medial support is an important factor in preventing major bone- and fracture-related postoperative complications such as reduction loss or nonunion.

## Background

In the operative treatment of unstable proximal humerus fractures, regardless of the type of fixation used, perioperative complications can occur, often leading to a worse clinical outcome than that obtained following nonoperative management. Especially in the patient with osteoporosis, there is no gold standard for surgical treatment, and the following complications have arisen following various treatment modalities: humeral head osteonecrosis, reduction loss, nonunion, malunion, plate breakage, screw problems, and infection [1-5].

The proximal humerus locking plate system has many advantages, including its anatomic design, low profile, the presence of suture holes, divergent angulation of locking screws, and high angular and rotational stability. It is believed that these locking plates provide improved fixation of proximal humerus fractures than the conventional plates, especially for bones that are osteoporotic.

Despite these advantages, it has been reported that locking plate technology for the surgical treatment of proximal humerus fractures has a high complication rate ranging from 16% to 36% [6-13]. Several major complications, such as reduction loss with or without screw perforation, nonunion, humeral head osteonecrosis, and metal breakage, may adversely affect clinical outcome.

Several studies [13-17] have stressed the importance of medial support in locked plating of proximal humerus fractures, and achieving mechanical support of the inferomedial region of the proximal humerus seems to be important for maintaining fracture reduction. We are aware of one report that identifies the clinical factors (i.e., medical co-morbidities, medial support, and head-neck-shaft angle) responsible for postoperative complications following the operative treatment of unstable proximal humerus fractures using a locking plate and suture augmentation.

We hypothesized that medial support decreases the complications of proximal humerus fractures treated with locking plate technology and suture augmentation, especially in older patients with osteoporosis. The objective of this study was to evaluate the role of medial support and clinical factors responsible on the outcomes and major complications associated with treatment of unstable proximal humerus fractures using a locking plate and suture augmentation.

## Methods

### Study design

A retrospective study was approved by the Institutional Review Board of Chonnam National University Hospital (CNUH) to analyze the clinical and radiographic outcomes of 62 patients (63 cases) who had been treated surgically for a proximal humerus fracture with locking plate fixation and tension band suture augmentation between September 2004 and October 2008. Preoperatively, the fracture pattern was determined according to the Neer classification [18] using a standardized preoperative surgical workup, which included conventional radiography, as well as 3-dimensional computed tomography (3D-CT) in all patients. Two fellowship-trained shoulder and elbow surgeons (ESM & MSK) used the same anatomic approach and surgical technique for all cases.

### Inclusion criteria

Adult patients who sustained a closed, unstable proximal humerus fracture without neurovascular complication at the time of injury and subsequently underwent operative management were identified for review. Those patients who were treated with an anatomic proximal humeral locking plate and suture augmentation were included in this study. The implants used in this series included 40 proximal humerus locking compression plates (PH-LCP, Synthes, Switzerland) and 23 PHILOS plates (Synthes, Switerland).

### Exclusion criteria

Adult patients were excluded from this study if it was determined that they sustained polytraumatic injuries or isolated proximal humerus fractures considered open, segmental or with diaphyseal extension. Patients with concomitant fractures of the ipsilateral elbow or distal radius were excluded from this study as well as those patients with nonunions, pathologic fractures, or refractures involving the proximal humerus.

### Operative technique and rehabilitation

Surgery was performed in a supine position on a radiolucent table, with side placement of an image intensifier to allow viewing of the humeral head in two planes. A standard deltopectoral approach was used in all patients. Fragment mobilization was achieved indirectly by placing nonabsorbable suture (# 2 FiberWire suture, Arthrex, Naples, FL) through the rotator cuff tendon adjacent to the tuberosity fragments. The sutures were used to assist in manipulation of the tuberosity fragments to allow for indirect reduction. A minimum of two sutures were placed in each case, with at least one placed in the substance of the infraspinatus tendon, and at least one in the subscapularis tendon. The medial cortex of the metaphyseal junction was reduced indirectly and the degree of reduction confirmed with an image intensifier. After confirming adequate reduction with an image intensifier, the achieved reduction was temporarily fixed with several 1.6-mm K-wires. When reduction was acceptable, a locking plate was placed inferior to the upper end of the greater tuberosity and lateral to the bicipital groove to avoid subacromial impingement. In fracture patterns where there was no inferomedial comminution and near anatomic reduction could be achieved, an oblique locking screw was not placed intentionally in the inferomedial quadrant of the proximal humerus. Conversely, in cases where inferomedial comminution was present, a reduction was of medial cortex was difficult, or the shaft could not be medialized and impacted into the head fragment, then we tried to place an oblique locking screw in the inferomedial quadrant of the proximal humerus to provide medial support.

Then suture augmentation in a tension band configuration was performed using the non-absorbable sutures that were passed through the rotator cuff tendons to secure the rotator cuff tendons to the holes in the locking plate. The surgical bed was irrigated with sterile normal saline and the soft tissue and skin were closed in a layered fashion.

After surgery, all shoulders were immobilized in a sling for the first 3 to 6 weeks, and passive range of motion exercise was allowed from 1 to 6 weeks postoperatively depending on fracture type, the degree of medial comminution, and intraoperative fixation stability. Elbow, wrist, and hand range of motion exercises were encouraged immediately. Stretching and resistive strengthening exercises were allowed when evidence of fracture healing was noted on follow-up radiographic imaging.

### Clinical and radiographic evaluation

Routine follow-up was initially performed at 3 weeks, 6 weeks, 3 months, 6 months, and 12 months postoperatively, and then annually. Clinical outcomes were determined using Neer’s evaluation criteria [18] at the final follow-up visit. Neer’s criteria include the following: pain (35 points), function (30 points), range of motion (25 points), and anatomy (10 points), with a total of 100 points. Outcomes on the Neer scale are based on total number of points: excellent is > 89; satisfactory is 80–89; unsatisfactory is 70–79; and failure is < 70.

Preoperative imaging of the injured shoulder was obtained to assist with surgical planning and consisted of three orthogonal radiographic views (anteroposterior, scapular Y, and axillary views), 3-D CT, and fluoroscopic evaluation of the shoulder in all patients. Postoperatively, we obtained serial follow-up radiographs (anteroposterior, scapular Y, and axillary views) at each visit. Preoperative and postoperative radiographs were retrospectively analyzed in a serial fashion by two independent observers (ESM and MSK) to evaluate the fracture type according to Neer’s classification, bony union, humeral neck-shaft angle, and complications.

We divided the cases into a medial support group (MS+ group) and a non-medial support group (MS- group) based on criteria suggested by Gardner et al. A fracture was considered to have medial support based on the following: (1) the medial pillar of the proximal humerus was anatomically reduced and not comminuted; (2) the shaft was medialized and impacted into the head fragment; (3) an oblique locking screw was placed directly into the inferomedial quadrant of the proximal humeral head fragment to within 5 mm of the subchondral bone [14].

Humeral neck-shaft angles were measured according to the Paavolainen method [19]. Briefly, a vertical axis was drawn from the superior border to the inferior border of the articular surface and a line perpendicular to this line was made that ran through the center of the humeral head. The angle between this perpendicular line and the line bisecting the humeral shaft was defined as the humeral neck-shaft angle. Postoperatively, restoration of humeral neck-shaft angle to 130° ± 10° was defined as good, fair was defined as being between 100° to 120° and poor was defined as being < 100°. Further, the presence of associated osteoporosis was measured by dual energy X-ray absorptiometry (DEXA) and defined when the patient had a T-score less than -2.5 involving the bone density of the hip and spine as established by the World Health Organization criteria for osteoporosis [20,21].

We evaluated the postoperative complications and categorized them into four groups [11]; (1) surgical technique-related complications; (2) soft-tissue- and wound-related complications; (3) implant- related complications; (4) bone- and fracture- related complications. Surgical technique-related complications attributed to the initial surgical procedure include primary screw perforation and plate irritation. Soft-tissue- and wound-related complications include superficial wound infection, deep wound infection, and neurological lesion. Implant-related complications include screw loosening and plate breakage [11]. Finally, bone- and fracture-related complications include reduction loss without screw perforation, reduction loss with screw perforation. Among these complications, we considered reduction loss with or without screw perforation, osteonecrosis of the humeral head, and nonunion, and metal breakage as major complications.

Overall clinical and radiographic outcomes as well as complications were evaluated and compared between the 2 groups. In addition, we analyzed the distributional pattern of complications based on the presence of medial support, the fracture type, and the presence of osteoporosis.

### Statistical analysis

Statistical analysis was performed using the SPSS software package (SPSS Statistics for Windows Release 17.0, Chicago, IL). All data were analyzed with use of Fisher’s exact test, Chi-square test, Independent-sample *t*-test, the Mann–Whitney *U* test, Kruskal-Wallis test, and Kendall’s tau-b correlation test. To identify the most influential clinical factors responsible for bone- and fracture- related complications, multivariate logistic regression analysis with combinations of three independent variables (the presence of medial support, preoperative fracture type, and the presence of osteoporosis) was performed. Finally, we performed the post-hoc power analysis (Statistics calculators, version 3.0). A p-value of <0.05 was considered statistically significant.

## Results

### Sociodemographic and clinical characteristics

There were 42 women and 20 men who underwent operative treatment of a proximal humerus fracture with locking plate technology and suture augmentation. One patient required open reduction and internal fixation for bilateral proximal humerus fractures. The average age was 62.2 years (range 18–92 years). The mean duration of follow-up was 13.4 months (range 12–38 months). The mechanism of shoulder injury was found to be a fall from a standing position in 33 patients, motor vehicle collision in 23 patients, and a fall from a height or stairs in seven patients. There were 42 two-part fractures, 18 three-part fractures, and three four-part fractures based on the Neer classification. The demographic data showed no significant differences in terms of age, sex, osteoporosis, or fracture pattern.

Review of the initial postoperative radiographs demonstrated that medial support was established in 36 cases according to Gardner’s criteria [14], while medial support was not reestablished in 27 cases. According to fracture type, there were 25 2-part fractures (69%), ten 3-part fractures (28%), and one 4-part fracture (3%) in the medial support group, while there were 17 2-part fractures (63%), eight 3-part fractures (30%), and two 4-part fractures (7%) in the non-medial support group (Table 1).

**Table 1:** Sociodemographic data and the clinical outcomes according to the presence of medial support

	**MS+ (n=36)**	**MS- (n=27)**	**Total (63)**	**Av. Neer’s score**	**P-value**
**Sex**	M: 9, F:27*	M:11, F:16*			0.184
**Age(yr)**	62.5	61.7	62.2		0.739
**Osteoporosis(%)**	20 (56)	16 (59)	36		0.769
**Fracture Type**					
**2 part**	25 (69)	17 (63)	42		
**3 part**	10 (28)	8 (30)	18		0.673
**4 part**	1 (3)	2 (7)	3		
**Neer criteria**					
**Excellent (>89)**	14 (38.9)	7 (25.9)	21 (33.3)		
**Satisfactory(80–90)**	15 (41.7)	10 (37.0)	25 (39.7)		
**Unsatisfactory(70–79)**	7 (19.4)	4 (14.9)	11 (17.5)		
**Failure(<70)**	0(0)	6 (22.2)	6 (9.5)		
**Av. Neer’s score**	85.7 ± 7.8	78.0 ± 14.2	82.4 ± 11.5		0.008
**Paavolainen Score**					
**Good (>130±10°)**	32 (88.9)	17 (62.9)	49 (77.8)	84.9 ± 8.3	
**Fair (100-120°)**	4 (11.1)	7 (26.0)	11 (17.5)	80.6 ± 9.9	0.004
**Poor(<100°)**	0 (0)	3 (11.1)	3 (4.8)	48.0 ± 6.1	
**Av. NSA**	130.4 ± 9.5	123.5 ± 21.3	127.4 ± 15.9		0.574

Values are given as number (percentage).

*one female have bilateral proximal humerus fractures.

MS+ = medial support group, MS- = non-medial support group, M = male, F = female, NSA = neck-shaft angle.

### Complications

There were 14 total complications in 13 of 63 cases noted during the follow-up period. There was one female patient who had two complications: (1) primary screw perforation during the index procedure, and (2) humeral head osteonecrosis at final follow-up (Table 2).

**Table 2:** The distributional pattern of complications according to the fracture types, the presence of medial support, and osteoporosis

**Complications (cases)**	**Fracture type**			**Medial support**		**Osteoporosis**		
	**2 part (42)**	**3 part (18)**	**4 part (3)**	**(+) (36)**	**(-) (27)**	**(+) (30)**	**(-) (33)**	**Total (63)**
**Screw perforation**	2^**†**^			2	0	2	0	2
**Plate irritation**			1	1	0	0	1	1
RL without SP*	2	1	2	0	5	4	1	5
RL with SP*		1		0	1	1	0	1
Nonunion*	1	1		0	2	1	1	2
**AVN of humoral head** *	1^**†**^			1	0	1	0	1
**Screw loosening**	1			0	1	0	1	1
Plate breakage*	1			0	1	0	1	1
**Total No. (%)**	**8 (19.0)**	**3 (16.7)**	**3 (100)**	**4 (11.1)**	**10 (37.0)**	**9 (30.0)**	**5 (15.2)**	**14 (22.2)**
**P-value**	**0.018**			**0.014**		**0.157**		

Values are given as number (percentage).

*Clinically serious major complication.

^**†**^One female patient had these two complications simultaneously.

RL = reduction loss, SP = screw perforation, AVN = avascular necrosis.

There were three surgical technique-related complications: primary screw perforation of the humeral head that was unrecognized during surgery in two cases and plate irritation due to high plating in one case. There were no soft-tissue/wound-related complications such as superficial wound infection and deep infection or neurological injury. There were nine bone- and fracture- related complications: five reduction losses without screw perforation; one reduction loss with screw perforation; two nonunions, and one humeral head osteonecrosis. Finally, there were two implant-related complications: one screw loosening and one plate breakage. Plate breakage occurred in a 58 year old male two months following the index procedure after he sustained a fall and landed on his outstretched, operative limb. This patient subsequently underwent osteosynthesis using a proximal humerus locking plate with iliac crest bone graft, which resulted in bony union. In the case of screw loosening, the screw was removed at postoperative day three and bony union was achieved by postoperative week twelve.

There were statistically significant differences in the overall incidence of complications based on the presence of medial support (p = 0.014) and the preoperative fracture type (p = 0.018), but no differences were noted in the presence of osteoporosis (p = 0.157).

Among 14 total complications, four (two screw perforation, one osteonecrosis, and one plate irritation) were seen in the medial support group, but only one was considered a major complication (humeral head osteonecrosis in an osteoporotic patient). The remaining ten complications were in the non-medial support group, nine of which were considered major complications (Table 2).

When we compared the nine major bone- and fracture- related complications, seven cases had osteoporosis, and six of these seven cases were in the non-medial support group. There were statistically significant differences in the overall incidence of complications based on the presence of medial support (p = 0.004) and preoperative fracture type (p = 0.035), but no differences based on the presence of osteoporosis (p = 0.073), sex (p = 0.301), or age (R^2^ = 0.176, p = 0.095) (Table 3 and 4).

**Table 3:** The distributional pattern focusing on the fracture- and bone- related complication

**Complications (cases)**	**Fracture type**			**Medial support**		**Osteoporosis**		
	**2 part (42)**	**3 part (18)**	**4 part (3)**	**(+) (36)**	**(-) (27)**	**(+) (30)**	**(-) (33)**	**Total (63)**
**RL without SP**	2*	1*	2*	0	5*	4*	1*	5*
**RL with SP**		1*		0	1*	1*	0	1*
**Nonunion**	1*	1*		0	2*	1*	1*	2*
**AVN of humoral head**	1			1	0	1	0	1
**Total No.(%)**	**4 (9.5)**	**3 (16.7)**	**2 (66.7)**	**1 (2.8)**	**8 (29.6)**	**7 (23.3)**	**2 (6.1)**	**9 (14.3)**
**P-value**	**0.035**			**0.004**		**0.073**		

Values are given as number (percentage).

*non-medial support group.

RL = reduction loss, SP = screw perforation.

**Table 4:** The relationship of osteoporosis and medial support confining to the fracture- and bone- related complications

	**Medial support group (N=36)**	**Non-medial support group (N=27)**	**Total**
	AVN of humoral head* (1)	RL without SP* (4)	
**Osteoporosis (+)**		RL with SP* (1)	**7(23.3) **
		Nonunion* (1)	
**Osteoporosis (-)**		RL without SP* (1)	**2 (6.1)**
		Nonunion* (1)	
**Total No. (%)**	**1 (11.1)**	**8 (37.0)**	**9 (100)**
**P-value**	**0.004**		

Values are given as number (percentage).

*Clinically serious major complication.

RL = reduction loss, SP = screw perforation, AVN = avascular necrosis.

Multivariate logistic regression analysis demonstrated that the presence of medial support was the only factor predictive of major bone- and fracture- related complications (B = -2.761, p = 0.016). Preoperative fracture type (B = 1.011, p = 0.118) and the presence of osteoporosis (B = -1.618, p = 0.086) did not have a statistically significant correlation with bone- and fracture- related complications.

Finally, when we compared the incidence of bone- and fracture- related complications based on the presence of medial support among 30 patients with osteoporosis, the group with medial support restoration had fewer complications than the group with medial support failure (Table 4).

### Clinical outcomes

According to Neer’s evaluation criteria, 21 of 63 cases (33.3%) showed excellent results, 25 cases (39.7%) were satisfactory, 11 cases (17.5%) were unsatisfactory, and six cases (9.5%) were failures. The excellent or satisfactory clinical results accounted for 73.0% of the all cases.

**Figure 1:**
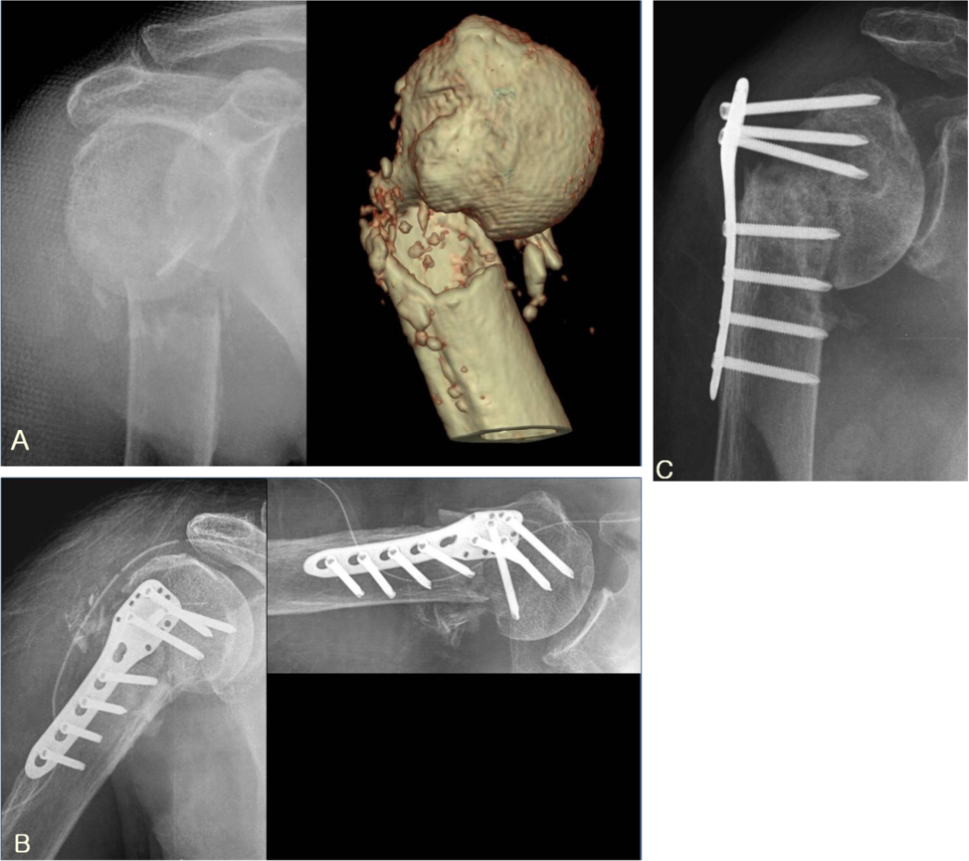
**(A) Initial radiographs of 82 years old male with osteoporosis showed 2 part proximal humerus fracture.** (**B**) Immediate postoperative radiographs showed fracture fixation in good alignment but medial support was not achieved. (**C**) At 4 months after operation, the reduction loss without screw perforation was developed. So, the clinical result was failure.

**Figure 2:**
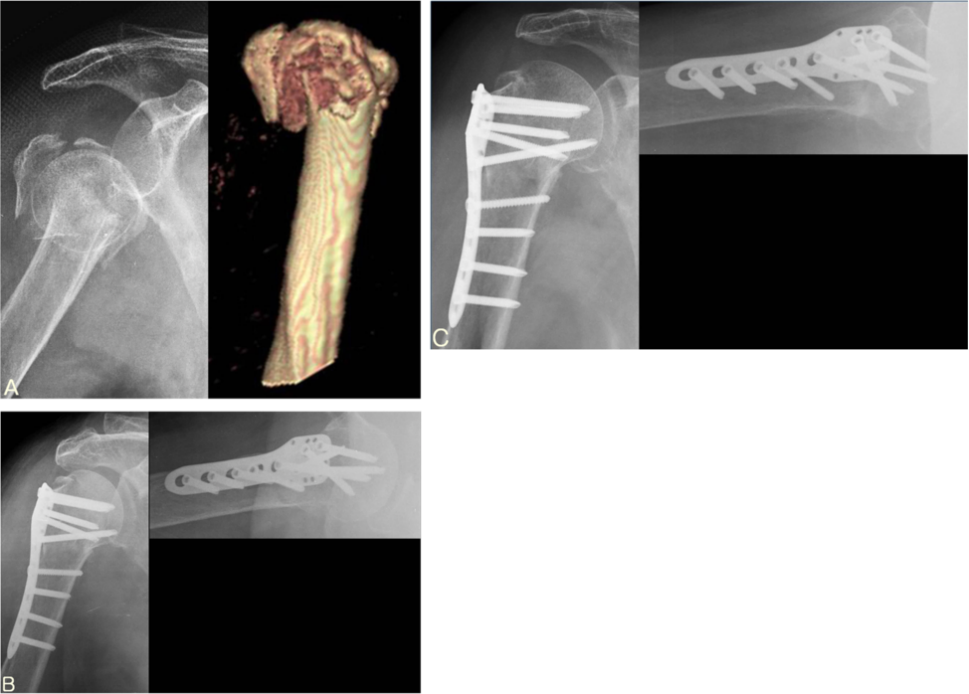
**(A) Initial radiograph of 82 years old female with osteoporosis showed 3 part proximal humerus fracture.** (**B**) Immediate postoperative radiograph showed good alignment and successful medial support was achieved by oblique long locking screw. (**C**) At 12 months follow up, radiograph showed complete bone union and good alignment and the clinical result was excellent.

Those six cases that went on to clinical failure included nonunion (N=2), humeral head osteonecrosis, reduction loss without screw perforation (Figure 1), reduction loss with screw perforation, and plate breakage. Twenty-nine cases (80.6%) showed excellent or satisfactory outcomes in the medial support group (36 cases) (Figure 2), while 17 cases (62.9%) demonstrated excellent or satisfactory outcomes in the non-medial support group (27 cases). The average Neer score of the medial support group was higher than that of the non-medial support group and this difference was statistically significant (MS+ group, 85.7 ± 7.8 (average ± SD); MS- group, 78.0 ± 14.2, (p = 0.008)) (Table 1).

### Radiographic outcomes

All fractures were united at final follow-up, except in four cases (two nonunions, one osteonecrosis, and one plate breakage). Forty-nine of the 63 cases (77.8%) showed good results by Paavolainen method, 11 cases (17.5%) had fair results, and three cases (4.8%) had poor results. Comparing neck-shaft angle according to the presence of medial support at last follow-up, 32 cases (88.9%) were scored excellent and four cases(11.1%) were scored fair. In the non-medial support group, 17 cases (62.9%) were scored excellent, seven cases (26.0%) were scored fair, and three cases (11.1%) were scored poor. There were no statistically significant differences in radiological outcome of neck-shaft angle between the two groups (MS+ group, 130.4 ± 9.5; MS- group, 123.5 ± 21.3, (p = 0.574)). However, according to the analysis of sub-group by Paavolainen method, The average Neer score of the poor group was lower than those of the Good or Fair group and this difference was statistically significant (Good, 84.9 ± 8.3 (average ± SD); Fair, 80.6 ± 9.9); Poor, 48.0 ± 6.1, (p = 0.004)) (Table 1).

## Discussion

Although the optimal surgical treatment of proximal humerus fractures has not been determined, there have been many operative techniques described, including percutaneous fixation, conventional plate fixation, intramedullary fixation with rods or pins, tension band wiring, and blade plate fixation, whose clinical outcomes have varied [12]. The current trend for treating these fractures utilizes locking plate technology as its lower profile may reduce impingement; its multiple divergent locking screw positions allow for improved fixation; and its biomechanical properties provide improved stability and load to failure. A recent biomechanical analysis in which blade-plate fixation was compared with locking plate fixation for the treatment of proximal humeral fractures demonstrated potential advantages with use of the locking plate technology [22].

In the current study, excellent or satisfactory clinical results were realized in 73.0% of cases, with good radiographical results in 77.8% of cases. These clinical and radiographic findings suggest that locking plate fixation and suture augmentation for proximal humerus fractures provide value as an operative treatment modality. However, room for improvement exists as 14 complications occurred in thirteen of 63 cases, representing a complication rate of 22%. These complications included screw perforation, plate irritation, loss of reduction, osteonecrosis, nonunion, screw loosening, and implant breakage (Table 2).

There have been several reports about the complications encountered with locking plate technology. Egol et al. [8] reported complications in 12 of 51 patients (24%) following proximal humerus locking plate fixation at 16 months follow-up. The complications occurred in eight patients (16%), including intraarticular screw penetration, osteonecrosis, acute fracture, nonunion, and heterotopic ossification. Similarly, Owsley et al. [10] reported a 36% complication rate in 53 patients, with intraarticular screw penetration occurring in 23% and a statistically significant higher radiographic complication rate noted in patients older than 60 years of age. In a study by Lee et al. [9], 20% of 45 patients had postoperative complications that included loss of fixation, adhesive capsulitis, and deep infection, while Sudkamp et al. [11] reported various complications in 34% of 155 patients including: screw penetration, plate impingement, infection, loss of reduction with or without screw perforation, humeral head osteonecrosis, nonunion, screw loosening, plate pullout, and implant breakage. Brunner et al. [7] reported an overall complication rate of 35% and Badman et al. [12] presented 13 complications (16%) in 81 patients and reported varus collapse in 5 patients (6%), intraarticular screw penetration in 3 (3.7%) and osteonecrosis in 5 (6.2%). Königshausen et al. [13] reported 12 (23.1%) complications in 73 patients. The overall complication rate in the current study was not higher compared to previous reports (22% versus 16- 36%)

Sudkamp et al. [11] classified the complications related to the locking plate into four categories. They reported 25 (40%) initial incorrect surgical technique-related complications amongst 62 total complications, which included primary intraoperative screw perforations of the humeral head in 21 cases and subacromial impingement due to significant cranial positioning of the plate in 4 cases. They suggested that adherence to proper surgical technique is necessary to avoid iatrogenic errors. Others, including Badman et al. [12] and Brunner et al. [7], have reported on primary and secondary intraarticular screw penetration into the glenohumeral joint and recommended that more accurate screw length measurement and shorter screw selection would prevent primary screw perforation.

In the current study, there were three (4.8%) initial incorrect surgical technique-related complications encountered in 63 cases at the end of the operative procedure: primary intraoperative screw perforation in two cases and subacromial impingement in one case. We believe that confirming screw position in more than one plane using an image intensifier will decrease the incidence of these surgical technique-related complications.

Of the four types of complications, we believe that bone- and fracture- related complication are the most important to prevent because they can negatively impact clinical and radiographic outcomes. More importantly though, this type of complication is under the surgeon’s control, and thus can be avoided with meticulous surgical technique. In the current study, all six cases with failed clinical outcomes had bone- and fracture- related complications. Our findings mirror previous work in this field, including Sudkamp et al. [11] who reported twenty one (13.5%) bone- and fracture- related complications in 155 patients.

The secondary screw perforation with reduction loss in the treatment of proximal humerus fractures with locking plates has already been described. The rigidity of this angled locking device is responsible for screws cutting through osteoporotic bone, leading to humeral head subsidence because of a deficient posteromedial bone buttress or osteonecrosis [7]. According to Brunner et al. [7], secondary varus angulation was observed in five patients, where screws were placed in three of these patients to support the medial buttress. However, none of the five patients received tension band sutures between the rotator cuff and the plate to neutralize traction forces. Based on their observations, they suggested that traction forces from the rotator cuff should be neutralized using tension band sutures combined with screws supporting the medial calcar, especially when medial support is insufficient. Similarly, Badman et al. [12] documented that restoration of the medial calcar and supplemental suture fixation may decrease the incidence of hardware-related complications.

In our study, augmentation with a tension band construct using non-absorbable sutures through the rotator cuff to the holes in the plate was applied in all cases. However, despite augmentation with a tension band construct, there were six cases where secondary reduction loss with or without screw perforation occurred in patients without medial support (Tables 3 and 4).

The incidence of humeral head osteonecrosis following locking plate fixation at short-term follow-up has been reported in 3.8% to 25% of cases [6,7,11,12]. In our current study, we had one case of humeral head osteonecrosis (1.6%) in a patient with a two-part proximal humerus fracture-dislocation. It is thought that open reduction and internal fixation may increase the risk of osteonecrosis unless the medial capsular structures and metaphyseal bony attachments are maintained so as to preserve the humeral head blood supply [23]. We believe that we had a low incidence of osteonecrosis because we were able to utilize the locking plate system to indirectly reduce the fracture fragments and avoid additional soft tissue dissection and damage near the fracture site.

We experienced plate breakage in one case, when a patient fell and landed on his outstretched, operative limb at two months following his index procedure. The patient subsequently underwent osteosynthesis using a proximal humerus locking plate with iliac crest bone graft, leading to successful bony union. We believe this complication was due to implant fatigue failure and a deficient posteromedial calcar in the setting of a low energy traumatic event. We believe that medial support may be important to resist implant fatigue.

Recently, much attention has been paid to the importance of the medial column for maintaining stable fixation of proximal humerus fractures [9,13-17,24-27]. Anatomic reduction and restoration of the medial calcar allow the medial column to both buttress and reduce the stresses of laterally-based plate fixation. Gardner et al. [14] first emphasized this concept by noting that when mechanical support of the inferomedial region of the proximal humerus was obtained, fracture subsidence was significantly reduced postoperatively. They suggested that mechanical support of the medial column may be achieved either with placement of humeral head screws inferomedially or endosteal fibular allograft strut augmentation when anatomic cortical contact is not possible [14,26,27]. They reported that lack of medial support led to a 30% screw perforation rate compared to a 6% screw perforation rate for fractures with an intact medial column.

According to our experience, direct placement of an oblique long locking screw into the inferomedial quadrant of the proximal humeral head is considered as the more important and substantive way to obtain the medial support when there is medial communition. In contrast, anatomical reduction of the medial calcar with good cortical contact, especially in patients without medial cortex comminution, may be additive in their ability to prevent postoperative complications.

Lee at al [9] reported that absence of comorbidity and the restoration of the medial metaphysis were the most reliable predictors of successful clinical outcomes, while Solberg et al. [24] recognized that the presence of a metaphyseal segment in the region of the medial calcar greater than 2 mm was associated with better clinical outcomes and independent of Neer fracture type. Others have corroborated the importance of restoring and maintaining the medial calcar to enhance mechanical stability and to avoid reduction loss [13,15,16,25]. In situations where the host bone is osteoporotic and anatomic reduction and restoration of the posteromedial column cannot be achieved, it is recommended that augmentation with endosteal fibular allograft struts or primary arthroplasty be considered [16]. Zhang et al. [15] reported clinical and radiological outcomes of seventy-two consecutive patients (mean 30.8-month follow-up, MS+ group: 29 patients; MS- group: 39 patients). They showed a statistically significant difference regarding the failure rate (23.1% in the MS- group vs. 3.4% in the MS+ group). They documented that the early loss of fixation was related to higher age and less initial neck-shaft angle of the patients. However, bone mineral density was not significantly associated with loss of fixation. They also observed a significantly lower final neck-shaft angle in the MS- group and greater secondary angle loss in the subgroup of Neer three-part (P = 0.033 and 0.015, respectively) and four-part fractures (P = 0.043 and 0.027). Therefore, they concluded that medial support for proximal humerus fractures seems to have no benefits in Neer two-part fractures, but the additional medial support screws inserted into the inferomedial region of the humeral head may help to enhance mechanical stability in complex fractures and allow for better maintenance of reduction.

In the current study, eight major bone- and fracture-related complications occurred in the non-medial support group, while one major bone- and fracture-related complication occurred in the medial support group. Utilizing regression analysis, we found that only one factor, namely the presence of medial support (and not preoperative fracture type), was responsible for predicting major bone- and fracture-related complications in the treatment of proximal humerus fractures using a locking plate and suture augmentation. There appears to be a trend towards significance for osteoporosis on multivariate regression analysis with p-values approaching 0.05. Further, a subgroup analysis of 30 cases with osteoporosis demonstrated that medial support restoration led to significantly less major bone- and fracture-related complications compared to medial support loss. Thus, even osteoporotic patients can benefit from achieving and maintaining medial support so they decrease their chances of having a major complication such as reduction loss or nonunion.

The limitations of the current study are its retrospective nature and small sample size, potentially introducing bias and β-error. We did not perform a priori power calculation analysis for sample size estimation as this work is a preliminary report of our experience. A post-hoc power analysis demonstrated that the observed power for the addition of the set of independent variables was 0.64458902. (Statistics calculators, version 3.0). Future work will focus on performing a prospective, randomized study that is adequately powered to have sufficient sample size to detect differences between the two groups. There was no control group in the present study; therefore, we cannot determine if another treatment method would have led to different results. In addition, we used two different types of locking plates (PH-LCP and PHILOS plate). These plates differ in profile and plate design, including the number of locking holes and the thickness of the plate, both of which can affect the overall outcome. Although we tried to place the oblique long locking screw into the inferomedial quadrant of the proximal humeral head intentionally after noticing the role of the medial support reported by Gardner et al. [14], some size mismatching between the humerus and the locking plates precluded the placement of the oblique long locking screw at the intended location. This led to both the MS+ and MS- groups comprising cases treated with both different types of locking plates, which can affect the overall outcome also. With regards to the presence or absence of osteoporosis, regional osteoporosis in the hip or spine was used in this study as a surrogate for the presence of local osteoporosis at the proximal humerus. But, this may be not the ideal method to assess local osteoporosis of the proximal humerus. Additionally, we divided the patients to two groups based on the presence or absence of osteoporosis (i.e., t-score < -2.5). We did not see any linear correlations between the t-scores and the other possible factors. Finally, although we describe a generalized postoperative rehabilitation protocol in the Methods, most cases had an individualized protocol for length of sling immobilization and timing of passive range of motion exercises. This depended on the fracture type, degree of medial comminution, and intraoperative fixation stability. These factors may have affected the clinical and radiographic outcomes.

## Conclusions

In the treatment of unstable proximal humerus fractures, locking plate technology and suture augmentation are considered a useful treatment modality based on adequate clinical and radiographic outcomes. We found that the presence of medial support is a critical, modifiable factor that the surgeon can control in preventing major complications. Therefore, we suggest that restoring medial support is important in preventing major bone- and fracture-related complications such as reduction loss or nonunion in the operative management of unstable proximal humerus fractures.

## Competing interests

The authors declare that they have no competing interests.

## Authors’ contributions

MSK designed this study, review the literatures and drafted the manuscript. JWB have made substantial contributions to acquisition of data, analysis and interpretation of data. ESM, SKK and DK have been involved in drafting the manuscript and revising it critically for important intellectual contents. All authors read and approved the final manuscript.

## Pre-publication history

The pre-publication history for this paper can be accessed here:

http://www.biomedcentral.com/1471-2474/14/102/prepub
